# Academic Resilience in Contexts of Inequality: Motivational and Self‐Efficacy Profiles of Disadvantaged High Achievers

**DOI:** 10.1002/jad.70190

**Published:** 2026-05-28

**Authors:** Margot Rémeau, Gaël Raffy, Grégoire Borst

**Affiliations:** ^1^ Université Paris Cité, LaPsyDÉ, CNRS Paris France; ^2^ Direction de l'évaluation, de la prospective et de la performance (DEPP) ministère de l’Éducation nationale Paris France; ^3^ Institut Universitaire de France Paris France

**Keywords:** academic resilience, achievement motivation, early adolescence, lower secondary education, self‐beliefs, socioeconomic inequality

## Abstract

**Introduction:**

Socioeconomic disparities in academic achievement are well‐documented in France and emerge early in schooling. Yet, a subset of students from socioeconomically disadvantaged backgrounds attains high academic performance, challenging deterministic accounts of educational inequality. Academic resilience offers a useful framework to identify these students, but little is known about the socio‐emotional characteristics that distinguish resilient learners during early adolescence, a critical developmental period.

**Methods:**

Using data from a nationally large cohort of 8934 French students (mean age = 11.50 years; 51% girls), we adopted a definition‐based approach to academic resilience. Learner profiles were constructed by cross‐classifying tertiles of family socioeconomic status (SES) and academic achievement assessed in Grade 7, yielding nine SES × achievement profiles. Intrinsic motivation, extrinsic motivation, and academic self‐efficacy were measured via student self‐report in Grade 6. Bayesian analyses of covariance were conducted to compare socio‐emotional profiles across groups, controlling for age and sex.

**Results:**

Decisive evidence emerged for differences in intrinsic motivation and self‐efficacy across learner profiles. Academically resilient students (low SES, high achievement) reported substantially higher intrinsic motivation and self‐efficacy than disadvantaged low achievers, and levels statistically indistinguishable from socioeconomically advantaged high achievers. Self‐efficacy showed the strongest differentiation across profiles and the steepest gradient across achievement levels. In contrast, extrinsic motivation exhibited weaker and less consistent differences between groups.

**Conclusions:**

These findings indicate that academic resilience in early adolescence is characterized by socio‐emotional profiles marked by strong self‐efficacy and elevated intrinsic motivation, comparable to those of advantaged high achievers. By highlighting psychological resources that differentiate resilient from non‐resilient students under conditions of socioeconomic disadvantage, this study identifies promising targets for interventions aimed at reducing educational inequalities.

## 1 Introduction

In education systems characterized by persistent socioeconomic disparities, the phenomenon of academic resilience, defined as performing at a high level despite socioeconomic disadvantage, challenges deterministic models of educational achievement (Wang et al. 1997). Students who demonstrate academic resilience are those who perform well at school despite facing an increased risk of academic failure associated with socioeconomic disadvantage, such as low family socioeconomic status (SES). Various methods are used in the literature to study this phenomenon (Rudd et al. 2021). Among these, the definition‐based approach explicitly identifies a sub‐sample of resilient students and compares them to a group of non‐resilient peers facing similar socio‐economic constraints, in contrast to variable‐centered or model‐based approaches that conceptualize resilience as a continuous or latent construct (Rudd et al. 2021). By directly contrasting students exposed to comparable levels of socioeconomic risk but exhibiting divergent academic outcomes, the definition‐based approach enables theoretically grounded and empirically transparent comparisons of cognitive and socio‐emotional characteristics. According to this theoretical framework, resilient students are defined as members of groups at elevated risk of low educational attainment who nonetheless achieve high levels of academic performance. For instance, in the Programme for International Student Assessment (PISA), resilient students are defined as those in the bottom quartile of the national socioeconomic distribution who achieve scores in the top quartile of academic performance (Schneider et al. 2018). Whereas some alternative approaches combine indicators of socioeconomic risk and academic performance into a single construct, the definition‐based approach clearly distinguishes exposure to disadvantage from academic outcomes by jointly but explicitly defining low SES and high achievement. This distinction allows for more precise comparisons between disadvantaged students who succeed academically and those who do not, thereby facilitating the identification of factors associated with academic success under conditions of socioeconomic constraint. This perspective is particularly informative in educational contexts characterized by persistent inequalities.

SES‐related educational inequalities are a global issue, as evidenced by meta‐analyses conducted across diverse national contexts (Kim et al. 2019; Liu et al. 2020; Selvitopu and Kaya 2023; Sirin 2005). Although international assessment data indicate that France is among the OECD countries with the most pronounced educational inequalities related to family SES (OECD 2023), French data are largely absent from existing meta‐analyses. Yet, evidence shows that SES‐based disparities in France emerge as early as the compulsory school entry age of three (Cioldi et al. 2025), and continue to widen throughout primary and secondary education (Delarue et al. 2024; Murat 2024). More advantaged students not only begin secondary school with stronger academic skills but also show faster progress in both mathematics and language compared to their less advantaged peers (Delarue et al. 2024). Specifically, by age 15, SES explains 21% of the variance in mathematics achievement and 17% in reading outcomes (OECD 2023). Understanding the mechanisms that promote academic resilience is therefore particularly critical in the French context, where early and persistent SES‐related inequalities call for evidence‐based strategies to support the success of disadvantaged students.

Despite growing interest in academic resilience, limited research has explored the socio‐emotional characteristics that may distinguish resilient students from their non‐resilient peers in the French educational context. Among the potential factors, motivation and self‐efficacy have been identified because they are consistently associated with academic success (Basileo et al. 2024; Haidari et al. 2023; Qi et al. 2024; Steinmayr and Spinath 2009). Self‐efficacy reflects students' belief in their ability to perform a specific academic task (Bandura and Wessels 1997). This belief extends beyond general confidence and is grounded in four primary sources: mastery experiences, vicarious learning, verbal persuasion, and individuals' physiological and emotional states (Bandura and Wessels 1997). High self‐efficacy fosters perseverance in the face of challenges, such as when a student enrolls in an advanced course despite its complexity, because they believe in their ability to learn. Conversely, low self‐efficacy can lead to avoidance or early withdrawal. Motivation can be divided into two dimensions according to self‐determination theory: intrinsic and extrinsic motivation (Deci and Ryan 1985; Ryan and Deci 2020). Intrinsic motivation emerges from a genuine interest in learning, while extrinsic motivation depends on external rewards or social pressures. However, this dichotomy is not absolute: extrinsic motivation can become autonomous if the student integrates the value of the task. Conversely, intrinsic motivation can be eroded in controlling contexts (Deci and Ryan 1985; Ryan and Deci 2020). A strong sense of self‐efficacy enhances intrinsic motivation by aligning effort with perceived competence, thereby promoting sustained academic engagement (Bandura and Wessels 1997; Schunk and DiBenedetto 2020). Extrinsic rewards, however, have dual effects: when framed as informational, they reinforce self‐efficacy and autonomy; when perceived as controlling, they externalize motivation, diminishing intrinsic curiosity (Deci et al. 1999; Ryan and Deci 2020).

Importantly, adolescence is a key developmental period for both academic trajectories and socio‐emotional development. This stage is marked by profound cognitive, motivational, and socio‐affective changes, including heightened sensitivity to social contexts, increasing autonomy, and identity exploration (Eccles and Roeser 2011; Yeager et al. 2018). These developmental transitions coincide with increasing academic demands and social comparisons in secondary education, rendering early adolescence a particularly informative window for understanding why some students succeed academically despite socioeconomic adversity.

From an academic resilience perspective, early evidence showed that learning motivation can help children overcome cumulative risk factors (Alfaro et al. 2009) and that intrinsic motivation explains a larger share of variance in reading performance among low‐achieving students than among their higher‐achieving peers (Logan et al. 2011). More recent studies extend these findings by demonstrating that, among students from low‐SES backgrounds, intrinsic motivation and academic self‐efficacy are robust predictors of academic achievement and engagement, and often mediate the association between socioeconomic disadvantage and school outcomes (Avci 2022; Rakesh et al. 2024; Steinmayr and Spinath 2009; Yan and Gai 2022). Furthermore, recent evidence suggests that some factors close to motivation and self‐efficacy may play a role in resilient trajectories. For example, Holzer et al. (2025) found that resilient achievers reported higher school happiness, better working memory, and greater metacognitive knowledge than underachievers, as well as a more positive academic self‐concept than both underachievers and expected low achievers, underscoring the joint contribution of cognitive and motivational resources to resilient trajectories.

However, despite adolescence being a period during which motivational orientations and self‐beliefs become increasingly salient for academic engagement and long‐term outcomes, few studies have explicitly examined how motivation and self‐efficacy underpin academic resilience during this developmental window, particularly in France, where inequalities appear earlier and persist more strongly than in many other OECD countries (Delarue et al. 2024; OECD 2023). Placing the focus on early adolescence is therefore especially relevant, as this period represents a turning point in both academic trajectories and the consolidation of socio‐emotional resources. Understanding resilience at this stage can thus offer critical insights into how some students sustain high performance under conditions of adversity, during a time of heightened developmental vulnerability and opportunity (Eccles and Roeser 2011; Yeager et al. 2018).

Given (a) the early and persistent impact of SES on academic achievement in the French context (Delarue et al. 2024), (b) the lack of studies investigating academic resilience using socio‐emotional variables during secondary education and in France, (c) the central role of motivation and self‐efficacy in supporting academic success (Basileo et al. 2024; Haidari et al. 2023; Qi et al. 2024; Steinmayr and Spinath 2009) and (d) the particular importance of socio‐emotional skills during adolescence (Eccles and Roeser 2011; Yeager et al. 2018), it is surprising that no study to date has investigated the profile of resilient students in the French system. In the present study, we did so by examining, among 8934 French students, whether intrinsic motivation, extrinsic motivation and self‐efficacy in Grade 6 differed according to the students' profiles in Grade 7, while controlling for age and sex, using Bayesian methods to quantify the strength of evidence for group differences. Based on previous studies showing that cognitive and socio‐emotional skills contribute to academic success under unfavorable conditions (Alfaro et al. 2009; Holzer et al. 2025; Logan et al. 2011), we hypothesized that resilient students in Grade 7 would display higher levels of intrinsic motivation and self‐efficacy in Grade 6 than disadvantaged low‐achieving students. We further hypothesized that resilient students in Grade 7 would exhibit levels of intrinsic motivation and self‐efficacy in Grade 6 comparable to those of privileged high‐achieving students, reflecting their academic success despite socio‐economic adversity. Finally, we examined whether extrinsic motivation in Grade 6 exhibited a similar pattern across learner profiles in Grade 7. Prior research suggests that, compared to intrinsic motivation and self‐efficacy, extrinsic motivation shows weaker and more heterogeneous associations with academic achievement, with findings that are less consistent across studies and contexts (Areepattamannil et al. 2011; Lemos and Veríssimo 2014; Vansteenkiste et al. 2020).

## 2 Methods

### 2.1 Participants

We used data from a nationally representative panel conducted by the statistical department of the French Ministry of Education (Direction de l’Évaluation, de la Prospective et de la Performance). This panel encompasses 15,188 students who were tracked from first grade (2011) through tenth grade (2020) in 919 schools in various geographic (urban, suburban, rural) and education setting (public, private and priority education zones). In the context of the present study, intrinsic and extrinsic motivation, and self‐efficacy were assessed in Grade 6, whereas academic achievement in numeracy and literacy was measured in Grade 7, allowing for a clear temporal ordering between predictors and subsequent academic outcomes. These school years represent a critical academic transition to secondary education and a formative stage in social‐emotional development (Evans et al. 2018). Thus, a subsample of 8934 students was selected based on the availability of data for both academic achievement measures (numeracy and literacy) and both indicators of family SES (guardian education level and occupation status). All sample characteristics are presented in Table 1. The sample includes 4568 girls (51.11%) and 4366 boys (48.89%), with a mean age of 11.50 years (SD = 0.30; range = 10.01−13.68). The study was approved by the National Council for Statistical Information (CNIS; visa n°. 2022A088ED) compliance with ethical standards, confidentiality requirements, and methodological rigor.

**Table 1:** Sample characteristics compared to the general population.

	Sample *N* = 8934		General population *N* = 828,881	
	*N*	%	*N*	%
*Sex*				
Girl	4568	51.11	407,218	49.13
Boy	4366	48.89	421,663	50.87
*School district*				
Private	1643	18.39	184,398	22.25
Public	6042	63.63	644,483	77.75
NA	1249	13.98	—	—

*Note:* Discrepancies between the study sample and the general population reflect both the study's inclusion criteria and data availability.

### 2.2 Materials and Procedure

Standardized digital assessments were administered in school settings to assess students' motivation and self‐efficacy in Grade 6, and numeracy and literacy in Grade 7. The assessments were conducted under the supervision of district advisors and school principals. Socio‐demographic information, including guardian education level and occupational status, was provided by legal guardians via a questionnaire in the fifth grade.

### 2.3 Academic Achievement

This study relied on academic achievement scores developed by the statistical department of the French Ministry of Education, using standardized and curriculum‐based tasks. These tasks were constructed through validated procedures and partially drawn from a national panel evaluation conducted in 1997 (Jeantheau and Murat 1998). The literacy component assessed various language skills, including comprehension, vocabulary (emphasizing lexical knowledge), grammar, spelling, and written expression. The numeracy component evaluated competencies in number operations, geometry, calculation, and problem‐solving. These academic scores were derived as psychometric measures within the broader framework of the panel study, using a vertical scaling procedure based on concurrent calibration and linear transformation to place student performance on a common metric across grade levels (for more information on scores, see Delarue et al. 2024). Items with inadequate psychometric properties, such as low point‐biserial correlations (< 0.20) or evidence of differential item functioning, were excluded to preserve the reliability and validity of the scales (Zumbo 2007). To preserve data integrity, students with over 50% missing responses in any given sequence were excluded from scoring (Kolen and Brennan 2014). Psychometric analyses indicated high internal consistency, with McDonald's omega coefficients of 0.95 for numeracy and 0.94 for literacy (Dunn et al. 2014).

*Score computation*. A composite indicator of academic achievement was constructed using Principal Component Analysis (PCA) with numeracy and literacy scores as input variables. The analysis identified a single dominant component that accounted for 86% of the total variance, with strong loadings from both numeracy and literacy (0.93 each). This component was standardized and included as a continuous variable in all subsequent analyses. This composite score captures students' overall academic achievement across the core domains of numeracy and literacy.

### 2.4 Family Socioeconomic Status

*Guardian education level*. The highest level of education attained by either guardian was used as the indicator of educational attainment within the household. This information was reported by legal guardians and converted into years of schooling on a standardized 12‐point scale, ranging from 1 (no formal education) to 12 (graduate degree).

*Occupational status*. Occupational status was assessed based on information provided by legal guardians, whose occupations were classified according to the PCS2020 system developed by the Institut national de la statistique et des études économiques (INSEE). Occupations were grouped into four categories: (1) highly advantaged (e.g., executives, company directors with 10 or more employees, higher intellectual professions, teachers); (2) advantaged (e.g., intermediate occupations, retired executives); (3) average (e.g., farmers, craftsmen, shopkeepers, employees); and (4) disadvantaged (e.g., blue‐collar workers, unemployed or inactive individuals). This classification is based on educational requirements and occupational positioning within the national labor market, rather than income levels. Each category was assigned a numerical value, with higher scores indicating higher occupational status. The highest occupational level reported by either guardian was used as the indicator of household occupational status.

*Score computation*. To construct a composite indicator of family SES, a PCA was performed using guardian educational level and occupational status as input variables (Oakes and Rossi 2003). The analysis yielded a single dominant component, explaining 81% of the total variance, with strong loadings from both education and occupation variables (0.90 each). This component was standardized and used as a continuous variable in subsequent analyses. This SES index captures structural and socio‐professional disadvantage, reflecting parental educational attainment and occupational positioning, which are closely associated with educational resources and employment stability but do not constitute a direct measure of material hardship; this operationalization aligns with approaches commonly used in large‐scale international assessments (e.g., OECD/PISA), while relying on nationally standard indicators in the French context (Avvisati 2020; Rémeau et al. 2026, 2026; Rémeau and Borst 2026).

### 2.5 Socio‐Emotional Skills

*Intrinsic motivation*. Four items were used to assess intrinsic motivation (see Table A1). Students rated each item on a four‐point Likert scale ranging from 1 (Strongly disagree) to 5 (Strongly agree). Items reflected students' interest in learning for its own sake, such as “I do my homework at home because I like to learn new things.” Internal consistency was good (*ω* = 0.83). A confirmatory factor analysis (CFA) supported the one‐factor structure, with adequate model fit: *χ* ^2^(17) = 1617.29, *p* < 0.001, RMSEA = 0.096, CFI = 0.94, TLI = 0.90. Covariances were added between items 1 and 2, and between items 2 and 4, to account for conceptual overlap (see Table A1). Items 1 and 2 both reflect intrinsic motivation across different settings (home and school), while items 2 and 4 highlight interest in learning as a source of personal satisfaction. All factor loadings were statistically significant at the 99.9% level.

*Extrinsic motivation*. Extrinsic motivation was measured using four items (see Table A2), rated on the same five‐point Likert scale. These items captured motivation driven by external demands or consequences, including statements such as “I try to do well at school, so that my teachers think I am a good student.” Internal consistency was acceptable (*ω* = 0.68). A one‐factor CFA confirmed the structure and showed adequate fit: *χ* ^2^(17) = 1617.29, *p* < 0.001, RMSEA = 0.096, CFI = 0.94, TLI = 0.90. All factor loadings were significant at the 99.9% level.

*Self‐efficacy*. Self‐efficacy was assessed with 10 items reflecting students' perceived ability to manage their academic tasks (see Table A3). Items were rated on a five‐point Likert scale from 1 (Strongly disagree) to 5 (Strongly agree), including examples such as “Are you able to concentrate on your schoolwork?” and “Are you able to motivate yourself for schoolwork?” Internal consistency was high (*ω* = 0.87). CFA results supported the unidimensional structure with excellent fit: *χ* ^2^(33) = 315.86, *p* < 0.001, RMSEA = 0.03, CFI = 0.99, TLI = 0.99. Covariances were added between items 4 and 5, and between items 9 and 10, based on theoretical and empirical considerations (see Table A3). Items 4 and 5 both relate to students' ability to manage and organize their schoolwork, while items 9 and 10 concern their perceived ability to meet adult expectations. All loadings were statistically significant at the 99.9% level.

### 2.6 Covariates

Age and sex were included as covariates in all statistical models. Sex was coded dichotomously (0 = boys, 1 = girls). Age was computed using students' birthdates relative to January 1st of the academic year in which they entered sixth grade. Both variables showed significant predictive associations with academic achievement (Givord 2020; Martinot et al. 2025; Voyer and Voyer 2014).

### 2.7 Statistical Analysis

All analyses were conducted using R (R. Core Team 2023). We applied a definition‐based approach to identify a subsample of resilient students (Rudd et al. 2021). To construct learner profiles, both the academic achievement index and the family SES index were divided into tertiles (low, middle, high), yielding nine SES × achievement profiles. All nine profiles were included in the analyses, allowing for a comprehensive examination of differences across the full distribution of SES and academic achievement. Next, we characterized the resulting profiles by examining demographic and socio‐emotional variables. Although differences across learner profiles are expected due to their construction, these analyses were conducted to formally establish overall group variation and to justify subsequent post hoc comparisons. One‐way analyses of variance (ANOVAs) and chi‐square tests were used to assess differences in age, sex distribution, family SES index, and academic achievement index across the profiles. When ANOVAs revealed significant effects, post hoc pairwise comparisons were conducted with Holm correction to adjust for multiple testing.

To examine group differences in intrinsic motivation, extrinsic motivation, and self‐efficacy while controlling for age and sex, we conducted Bayesian analyses of covariance (ANCOVAs) using the *BayesFactor* package (Morey et al. 2025). Learner profiles were included as fixed factors, with age and sex entered as covariates. Competing models were compared using Bayes Factors (BF_10_), allowing to quantify the evidence for the contribution of learner profiles over and above demographic covariates (Morey et al. 2025). Bayesian post hoc comparisons were conducted using pairwise *t*‐tests with Cauchy priors. Bayes Factors were used to interpret the strength of evidence for group differences. Finally, model‐averaged *R* ^2^ values were reported to quantify the proportion of explained variance, serving as a standardized effect size indicator within the Bayesian framework.

## 3 Results

### 3.1 Descriptive Analyses

As shown in Table 2, family SES was moderately and positively associated with academic achievement (*r* = 0.42, *p* < 0.001), indicating that higher socio‐economic background is associated to better academic performance. In contrast, the correlations between SES and socio‐emotional skills were weak: negative for intrinsic motivation (*r* = –0.02, *p* < 0.05), slightly positive for extrinsic motivation (*r* = 0.01, *p* < 0.01), and more substantial for self‐efficacy (*r* = 0.08, *p* < 0.001). Academic achievement, in turn, was positively associated with all three socio‐emotional variables, with small correlations for intrinsic and extrinsic motivation (both *r* = 0.07, *p* < 0.001), and a stronger association with self‐efficacy (*r* = 0.25, *p* < 0.001).

**Table 2:** Descriptive and bivariate correlation analyses for all variables.

Measures	1	2	3	4	5	6	7	8	9	10	11
*Individual backgrounds*											
1. Age	—										
2. Sex (girl = 1)	−0.03**	—									
3. Education level	−0.06***	−0.02*	—								
4. Occupation level	−0.03***	−0.02*	0.61***	—							
5. Family SES index	−0.05***	−0.02*	0.90***	0.90***	—						
*Academic achievement*											
6. Numeracy	0.01	−0.08***	0.34***	0.34***	0.38***	—					
7. Literacy	0.01*	0.18***	0.34***	0.34***	0.38***	0.67***	—				
8. Academic achievement index	0.00	0.06***	0.38***	0.37***	0.42***	0.91***	0.91***	—			
*Socio‐emotional skills*											
9. Intrinsic motivation	−0.01	0.03***	−0.01*	−0.01*	−0.01*	0.06***	0.06***	0.07***	—		
10. Extrinsic motivation	0.00	−0.02**	0.01*	0.01*	0.01*	0.06***	0.06***	0.06***	0.69***	—	
11. Self‐efficacy	0.02**	0.15***	0.07***	0.06***	0.07***	0.18***	0.23***	0.23***	0.56***	0.41***	—
*N*	8480	8934	8934	8934	8934	8934	8934	8934	8084	8084	8118
*M*	11.50	—	9.35	2.72	0	0	0	0	0	0	0
SD	0.30	—	2.83	1.01	1	1	1	1	1	1	1
*Skew*	0.08	—	−1.08	−1.08	−0.45	−0.06	0.22	0.05	−0.50	−0.47	0.05
*Kurtosis*	−0.18	—	0.31	−1.19	−0.71	0.13	0.22	−0.10	−0.50	−0.35	0.02
Missing %	5.08%	0%	0%	0%	0%	0%	0%	0%	9.51%	9.51%	9.14%

*Note:* Girl was coded in 1 and boy in 0.

### 3.2 Description of the Socio‐Demographic Characteristics

Learner profiles were defined by the cross‐classification of tertiles of family SES (SES: low, mid, high) and academic achievement (ACAD: low, mid, high), resulting in nine distinct groups (see Table 3). Global group differences were first examined using omnibus tests (see Table A4). A one‐way ANOVA revealed a highly significant effect of learner profile on the academic achievement index, *F*(1, 8) = 10,631.47, *p* < 0.001, *η* ^2^ = 0.803, indicating very large differences in academic performance across the nine profiles. Similarly, a strong effect of learner profile was observed for the family SES index, *F*(1, 8) = 17,560.04, *p* < 0.001, *η* ^2^ = 0.857, reflecting the expected socioeconomic stratification across groups. Significant global differences were also found for age, *F*(1, 8) = 2,006,777.55, *p* < 0.001, although the associated effect size was very small (*η* ^2^ = 0.003), suggesting minimal practical differences in age across profiles. Finally, a chi‐square test indicated a statistically significant association between sex and learner profile, *χ* ^2^(8) = 34.27, *p* < 0.001, with a small effect size (Cramer's *V* = 0.062). Inspection of group distributions indicated that girls were slightly overrepresented in higher‐achieving profiles, whereas boys were relatively more represented in lower‐achieving profiles, although these differences remained modest in magnitude.

**Table 3:** Description of distinguished groups.

Variable	SES low × ACAD low	SES low × ACAD mid	SES low × ACAD high	SES mid × ACAD low	SES mid × ACAD mid	SES mid × ACAD high	SES high × ACAD low	SES high × ACAD mid	SES high × ACAD high
*N*	1587 (17.8%)	927 (10.4%)	464 (5.2%)	978 (10.9%)	1085 (12.1%)	915 (10.2%)	413 (4.6%)	966 (10.8%)	1599 (17.9%)
Age (years)	11.51 (0.31)	11.5 (0.29)	11.52 (0.3)	11.46 (0.29)	11.5 (0.29)	11.51 (0.31)	11.47 (0.29)	11.49 (0.3)	11.49 (0.31)
Girls, n (%)	807 (50.9%)	468 (50.5%)	264 (56.9%)	475 (48.6%)	562 (51.8%)	498 (54.4%)	187 (45.3%)	444 (46%)	863 (54%)
Academic achievement index	−1.15 (0.54)	−0.05 (0.25)	0.94 (0.45)	−1.06 (0.49)	−0.02 (0.25)	0.99 (0.47)	−0.91 (0.41)	0.04 (0.25)	1.2 (0.57)
Numeracy in Grade 7	−1.09 (0.66)	−0.02 (0.45)	0.9 (0.59)	−0.97 (0.61)	0.01 (0.47)	0.9 (0.57)	−0.8 (0.55)	0.06 (0.46)	1.08 (0.63)
Literacy in Grade 7	−1.02 (0.59)	−0.08 (0.45)	0.82 (0.56)	−0.97 (0.57)	−0.04 (0.46)	0.91 (0.61)	−0.87 (0.52)	0.02 (0.47)	1.13 (0.71)
Family SES index	−1.26 (0.56)	−1.11 (0.51)	−1.04 (0.5)	0.06 (0.3)	0.1 (0.3)	0.15 (0.3)	1 (0.26)	1.07 (0.23)	1.11 (0.22)
Guardian education level	5.85 (2.35)	6.42 (2.27)	6.73 (2.25)	9.95 (1.44)	10.25 (1.3)	10.35 (1.27)	11.5 (0.75)	11.66 (0.59)	11.78 (0.51)
Occupational status: category 1	568 (35.8%)	285 (30.7%)	134 (28.9%)	8 (0.8%)	8 (0.7%)	8 (0.9%)	0 (0%)	0 (0%)	0 (0%)
Occupational status: category 2	943 (59.4%)	593 (64%)	306 (65.9%)	427 (43.7%)	482 (44.4%)	361 (39.5%)	0 (0%)	0 (0%)	0 (0%)
Occupational status: category 3	70 (4.4%)	42 (4.5%)	18 (3.9%)	485 (49.6%)	548 (50.5%)	511 (55.8%)	99 (24%)	171 (17.7%)	231 (14.4%)
Occupational status: category 4	6 (0.4%)	7 (0.8%)	6 (1.3%)	58 (5.9%)	47 (4.3%)	35 (3.8%)	314 (76%)	795 (82.3%)	1368 (85.6%)

*Note:* Values are reported as mean (standard deviation) for continuous variables and as n (%) for categorical variables. Academic achievement, numeracy, literacy, and family SES indices are standardized (*z*‐scores), with higher values indicating higher levels of achievement or SES. Learner profiles are defined by the cross‐classification of tertiles of family socioeconomic status (SES: low, mid, high) and academic achievement (ACAD: low, mid, high). Numeracy and literacy scores correspond to assessments conducted in Grade 7, whereas age, sex, and socio‐emotional characteristics were assessed in Grade 6. Guardian education level is reported on the original scale of the national classification. Occupational status categories refer to nationally defined socio‐professional groups (see Methods). Inferential statistics and tests of group differences are reported in Appendix Table A4, Cohen's *d* are reported in Figure A1 and group‐level distributions are represented in Figure A2.

**Figure A1:**
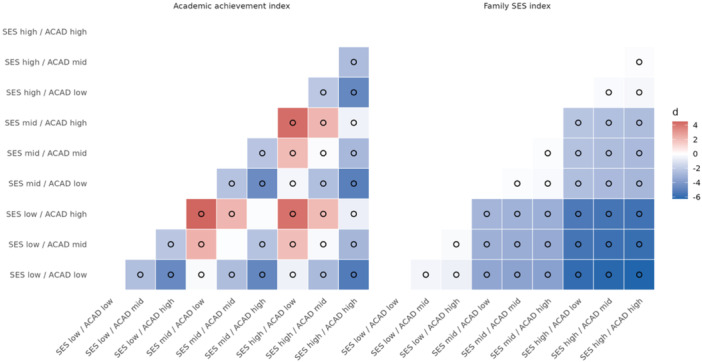
Pairwise comparisons of learner profiles on academic achievement and family SES indexes. Heatmaps display pairwise standardized mean differences (Cohen's *d*) between the nine learner profiles defined by the cross‐classification of family SES (SES: low, mid, high) and academic achievement (ACAD: low, mid, high). The left panel corresponds to the academic achievement index, and the right panel to the family SES index. Color intensity reflects the magnitude and direction of the effect size, with warmer colors indicating higher values for the row group relative to the column group and cooler colors indicating lower values. Dots indicate contrasts that remain statistically significant after Holm correction for multiple comparisons. Importantly, although significant but weak differences are observed among low‐SES profiles, all low‐SES groups remain within the lowest SES tertile, indicating that within‐tercile variation does not reflect access to higher socioeconomic strata.

**Figure A2:**
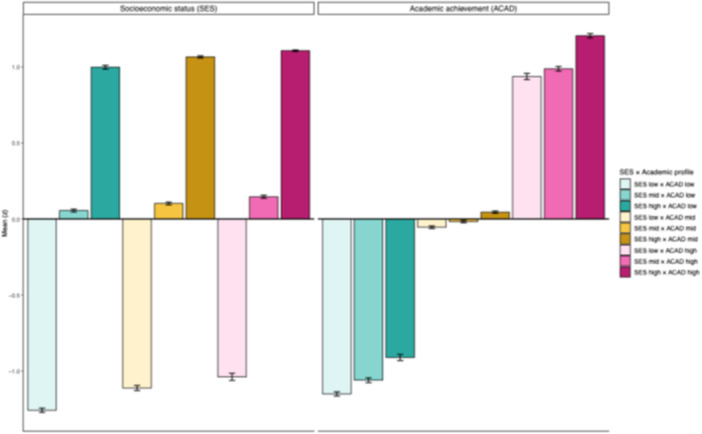
Mean family SES and academic achievement across learner profiles. Bars represent mean standardized scores (z‐scores) of family SES (SES; left panel) and academic achievement (ACAD; right panel) for each learner profile defined by the cross‐classification of SES tertiles (low, mid, high) and academic achievement tertiles (low, mid, high). Error bars indicate ±1 standard error of the mean. As expected from the construction of the profiles, SES varies primarily across SES tertiles and academic achievement varies primarily across achievement tertiles. Importantly, although small mean differences are observed between profiles within the same SES tertile, all such profiles remain clearly separated from adjacent SES tertiles, indicating that within‐tertile variation does not correspond to access to higher socioeconomic strata.

To clarify the structure of these omnibus effects, Holm‐adjusted post hoc comparisons were conducted for both indexes (see Figure A1 for Cohen's *d* and Figure A2 for group‐level distributions). For the academic achievement index, post hoc analyses revealed a clear and systematic hierarchy driven primarily by achievement level (see Figure A2). Across all SES strata, high‐achieving profiles scored significantly higher than mid‐ and low‐achieving profiles (all *p*s < 0.001), with very large effect sizes (*d*s typically > 2.0). Notably, low‐SES high achievers (academically resilient learners) significantly outperformed all low‐ and mid‐achieving profiles across SES strata (all *p*s < 0.001), including high‐SES low achievers. However, their academic achievement remained similar to that of high‐achieving students from mid‐SES backgrounds and lower than that of high‐SES high‐achieving students, indicating that socioeconomic advantage continues to confer an additional performance benefit even among high achievers. For the family SES index, post hoc comparisons showed a strong and monotonic socioeconomic gradient (see Figure A2). High‐SES profiles differed significantly from both mid‐ and low‐SES profiles across all achievement levels (all *p*s < 0.001), with extremely large effect sizes (*d*s frequently exceeding 3.0). Although statistically significant but weak differences emerged among low‐SES profiles, all such groups belonged to the lowest SES tercile by construction. These within‐tercile variations therefore, do not undermine the interpretation of low‐SES high achievers as succeeding under persistent socioeconomic disadvantage.

### 3.3 Description of the Socio‐Emotional Skills

**Figure 1:**
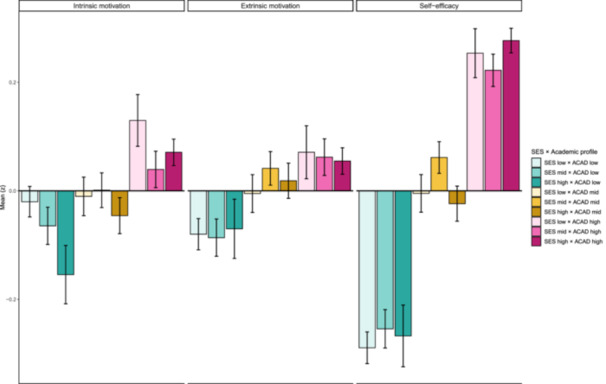
Socio‐emotional profiles across SES × academic achievement groups. Bars represent standardized mean scores (*z*‐scores) of intrinsic motivation, extrinsic motivation, and self‐efficacy across the nine learner profiles defined by the cross‐classification of family socioeconomic status (SES: low, mid, high) and academic achievement (ACAD: low, mid, high). Error bars indicate standard errors of the mean. Higher values indicate higher levels of the corresponding socio‐emotional construct. Socio‐emotional measures were assessed in Grade 6. Group differences were evaluated using inferential analyses reported in the Description of the socio‐emotional skills section.

For intrinsic motivation, Bayesian ANCOVA indicated that models including learner profiles provided a substantially better account of the data than models additionally including age and sex. There was moderate‐to‐strong evidence against the inclusion of age (BF_incl = 0.04) and sex (BF_incl = 0.38), indicating that these covariates did not contribute meaningfully beyond learner profiles. The model‐averaged proportion of explained variance was small but non‐zero (*R* ^2^ = 0.005, 95% CI [.002, 0.008]). Focusing on students from low socioeconomic backgrounds, Bayesian post hoc comparisons revealed strong evidence that low‐SES high achievers differed from their low‐SES peers with mid and low academic achievement (see Table A5 and Figure 1). Specifically, resilient students showed higher levels of intrinsic motivation than low‐SES low achievers and low‐SES mid achievers (BF_10_s > 1), indicating differences within the same level of socioeconomic disadvantage. In contrast, comparisons between low‐SES high achievers and other high‐achieving students from middle‐ or high‐SES backgrounds yielded little to no evidence for differences (BF_10_ = 1 or <1), suggesting that resilient students reached intrinsic motivational levels comparable to those of more socioeconomically advantaged high achievers.

For extrinsic motivation, the Bayesian ANCOVA provided no compelling evidence for a substantive effect of learner profiles, once age and sex were controlled. Model comparison results indicated that adding age or sex to the model did not improve explanatory power, with moderate evidence against the inclusion of age (BF_incl = 0.03) and against the inclusion of sex (BF_incl = 0.18). These findings suggest that demographic covariates did not contribute meaningfully to explaining individual differences in extrinsic motivation beyond learner profile membership. The overall proportion of variance explained by the model was very small (model‐averaged *R* ^2^ = 0.004, 95% credible interval [0.002, 0.007]), indicating that extrinsic motivation varied only marginally across socioeconomic and academic profiles. Bayesian post hoc comparisons further corroborated this pattern (see Table A6 and Figure 1). Focusing on students from low socioeconomic backgrounds, comparisons between academically resilient students (low SES × high achievement) and their low‐SES peers with low or mid academic achievement yielded only weak or ambiguous evidence for group differences in extrinsic motivation (BF_10_ values generally close to 1). Similarly, comparisons between low‐SES high achievers and high‐achieving students from middle‐ or high‐SES backgrounds consistently produced Bayes factors below conventional thresholds for moderate evidence (BF_10_ < 1), indicating little support for systematic differences between these groups.

For self‐efficacy, Bayesian ANCOVA provided decisive evidence for differences between learner profiles, beyond age and sex. Models including learner profiles were overwhelmingly favored over models additionally including covariates (BF_10_ > 10^35^). Examination of inclusion Bayes factors indicated extreme evidence for the inclusion of sex (BF_incl = 2.73 × 10^36^), whereas evidence for age was weak and favored exclusion (BF_incl = 0.32). The model‐averaged proportion of explained variance was substantial for a socio‐emotional outcome (*R^2^* = 0.064, 95% CI [.054, 0.075]), indicating that learner profiles captured a meaningful share of individual differences in academic self‐efficacy. Bayesian post hoc comparisons revealed a highly structured pattern aligned with academic achievement level (see Table A7 and Figure 1). Focusing on students from low socioeconomic backgrounds, low‐SES high achievers exhibited markedly higher self‐efficacy than their low‐SES peers with low academic achievement (BF_10_ ≈ 9.8 × 10^13^), providing decisive evidence for differences within the same level of socioeconomic disadvantage. In contrast, no meaningful differences emerged between low‐SES high achievers and high‐achieving students from middle‐ or high‐SES backgrounds (BF_10_s < 1), indicating comparable levels of self‐efficacy across high‐achieving profiles regardless of SES.

## 4 Discussion

This study examined whether resilient learners in Grade 7—defined as students from low‐SES backgrounds who belong to the highest tertile of academic achievement—differ from their peers in intrinsic motivation, extrinsic motivation, and self‐efficacy in Grade 6. Using a definition‐based approach applied to a nationally large French sample of 8934 students, we identified nine learner profiles resulting from the cross‐classification of family SES (low, mid, high) and academic achievement (low, mid, high). Our findings provide novel insights into the socio‐emotional characteristics that may support academic resilience in early adolescence, a developmental period marked by both academic transitions and critical changes in identity and motivation (Eccles and Roeser 2011; Steinberg 2014; Yeager et al. 2018).

Among the three socio‐emotional skills examined, self‐efficacy emerged as the most powerful discriminator across learner profiles. Bayesian analyses revealed decisive evidence for differences in self‐efficacy across profiles, with learner profiles explaining a substantial proportion of variance. Academically resilient learners reported markedly higher self‐efficacy than disadvantaged low‐achieving students, with decisive evidence supporting this contrast, and did not differ meaningfully from high‐SES high‐achieving students. This pattern supports our hypothesis and aligns with prior work identifying self‐efficacy as a robust correlate of academic achievement under adverse conditions (Bandura and Wessels 1997). More broadly, these findings underscore the central role of self‐efficacy as a protective factor, suggesting that resilient learners not only achieve high academic performance but also internalize strong beliefs in their capacity to succeed despite socioeconomic disadvantage. This interpretation is consistent with recent evidence showing higher academic self‐concept among resilient students relative to non‐resilient peers (Holzer et al. 2025). However, although self‐efficacy was assessed in Grade 6 and academic achievement in Grade 7, providing a clear temporal ordering between beliefs and subsequent performance, the observational nature of the design precludes causal inference. Accordingly, the present analyses cannot disentangle whether higher self‐efficacy facilitates later academic success or whether repeated academic success strengthens students' beliefs through accumulated mastery experiences.

For intrinsic motivation, a closely parallel but more modest pattern emerged. Bayesian ANCOVA indicated clear evidence for differences across learner profiles, although the proportion of explained variance was smaller. Post hoc comparisons showed that resilient learners reported higher intrinsic motivation than disadvantaged low‐achieving students, whereas intrinsic motivation levels among resilient learners were statistically indistinguishable from those of high‐SES high achievers, with Bayes factors favoring the null hypothesis. Across the full distribution of profiles, intrinsic motivation increased systematically with academic achievement within each SES tertile, whereas SES‐related differences within achievement levels were comparatively small and often supported by weak or inconclusive evidence. This pattern suggests that intrinsic motivation is more tightly aligned with academic success than with socioeconomic background per se. These findings are consistent with theoretical accounts emphasizing the internalization of learning goals among successful students, even in contexts of constrained resources (Steinmayr and Spinath 2009). As with self‐efficacy, although intrinsic motivation was measured prior to academic achievement, the correlational design does not allow causal conclusions, and reciprocal processes between motivation and achievement likely operate over time.

In contrast, extrinsic motivation showed substantially weaker and less consistent differentiation across learner profiles. Bayesian ANCOVA yielded limited evidence for systematic profile‐related differences, and the proportion of explained variance was minimal. Post hoc comparisons revealed no robust contrasts distinguishing resilient learners from either disadvantaged low achievers or advantaged high achievers, with most Bayes factors indicating weak evidence or evidence favoring the null hypothesis. Across profiles, extrinsic motivation displayed limited alignment with academic achievement and inconsistent associations with SES, suggesting that externally regulated forms of motivation play a comparatively minor role in differentiating resilient trajectories during early adolescence. This finding is consistent with prior research highlighting the heterogeneous, context‐dependent, and sometimes compensatory role of extrinsic motivation in academic functioning (Corpus et al. 2020; Linnenbrink‐Garcia et al. 2016; Vansteenkiste et al. 2020).

Importantly, the fine‐grained SES × achievement classification clarifies that socio‐emotional differentiation aligns more closely with academic achievement than with socioeconomic position. Within each SES tertile, high‐achieving students, regardless of their socioeconomic background, exhibited higher levels of self‐efficacy and intrinsic motivation than their lower‐achieving peers. Resilient learners clustered with high achievers rather than socioeconomically similar lower‐achieving peers, supporting the view of resilience as the alignment of strong performance and adaptive motivation. In addition, although resilient learners and socioeconomically advantaged high achievers exhibited comparable levels of self‐efficacy and intrinsic motivation, they did not reach identical levels of academic achievement. Socioeconomically advantaged high achievers remained the highest‐performing group overall, underscoring that motivational and self‐belief resources, while critical, are not sufficient on their own to fully close SES‐related achievement gaps. This persistent disparity suggests that structural advantages associated with higher SES confer additional academic benefits, likely through greater access to educational resources, enrichment opportunities, and cognitively stimulating environments, as well as stronger skills such as executive functions and metacognition (Delarue et al. 2024; Holzer et al. 2025; Rakesh et al. 2024; Rémeau and Borst 2026; Sirin 2005). Moreover, cumulative exposure to stress, food or housing insecurity, and other contextual constraints associated with lower SES may impose cognitive and emotional demands that can hinder academic performance, despite the presence of positive motivational attributes (Blair and Raver 2016; Evans and Kim 2013). Thus, the achievement gap reflects both individual psychological characteristics and broader material, social, and institutional resources shaping development and learning (Bronfenbrenner 1996). In sum, resilience reflects the presence of socio‐emotional strengths, especially self‐efficacy and intrinsic motivation, comparable to those of advantaged high achievers (Martin and Marsh 2009; Wang et al. 1997).

From a theoretical perspective, these results support integrative models of academic resilience that emphasize the joint contribution of cognitive, motivational, and contextual factors (Martin and Marsh 2009). The convergence of resilient learners and advantaged high achievers in self‐efficacy and intrinsic motivation suggests that these socio‐emotional resources may partially offset the disadvantages associated with low SES, enabling students to sustain high levels of academic engagement and performance. This finding is particularly informative in the French context, where SES‐related educational inequalities are both early and persistent (Delarue et al. 2024; OECD 2023).

From a practical perspective, the findings suggest that interventions targeting self‐efficacy and intrinsic motivation may be especially effective in supporting disadvantaged students. School‐based programs that promote academic confidence, autonomy‐supportive teaching practices, and mastery‐oriented learning environments have been shown to enhance both academic outcomes and socio‐emotional well‐being (Yeager et al. 2018). Crucially, the present results also indicate that socioeconomic advantage alone does not guarantee strong motivational resources: low‐achieving students from higher‐SES backgrounds consistently reported lower levels of self‐efficacy and intrinsic motivation, which may partly account for their underachievement. These findings underscore the importance of identifying and supporting motivational vulnerabilities across the full socioeconomic spectrum and argue for differentiated intervention strategies that move beyond SES as a sole marker of risk (Luthar et al. 2020).

Several limitations should be acknowledged. First, although Bayesian inference allowed for a nuanced quantification of evidence strength, the observational design precludes causal conclusions regarding the directionality of associations between socio‐emotional skills and academic achievement. Longitudinal analyses are needed to examine how self‐efficacy and motivation evolve over time and whether they prospectively predict resilient trajectories. Second, although learner profiles were defined using SES tertiles, some within‐tertile variation in SES remained across profiles. As shown in Table 3, SES tends to increase gradually across profiles, even within the same tertile, reflecting the continuous nature of the SES composite index. This suggests that profiles classified within the same SES category may still differ to some extent in their level of socioeconomic advantage. While the tertile‐based classification ensures that all groups fall within the same broad SES range, it is possible that residual variation may modestly contribute to the observed differences in socio‐emotional skills and academic achievement. Accordingly, the present analyses do not fully isolate the independent contribution of SES from that of learner profile membership. Future research could complement this approach by modelling SES as a continuous variable to further examine its role in shaping these associations. Third, although psychometrically robust measures were used, socio‐emotional skills were assessed via self‐report, which may be subject to social desirability or comprehension biases in early adolescence (Duckworth and Yeager 2015; OECD 2023). Future research should incorporate additional cognitive and contextual indicators—such as executive functions, metacognition, emotion regulation, and family or school support—and examine how these factors jointly shape resilience over time (Luthar et al. 2020; Skinner 2023).

In conclusion, this study provides robust evidence that academically resilient learners in France in Grade 7—defined as low‐SES students who achieve at high levels—exhibit socio‐emotional profiles characterized by strong self‐efficacy and elevated intrinsic motivation in Grade 6, comparable to those of socioeconomically advantaged high achievers. These findings highlight the central role of motivational and self‐belief processes in enabling students to overcome structural disadvantage and point to promising leverage points for policies and interventions aimed at reducing educational inequalities through the joint support of academic and socio‐emotional development.

## Author Contributions

**Margot Rémeau:** conceptualization, investigation, writing – original draft, methodology, visualization, writing – review and editing, software, formal analysis, project administration, data curation, resources. **Gaël Raffy:** data curation, resources, validation, writing – review and editing. **Grégoire Borst:** writing – review and editing, supervision.

## Ethics Statement

The study was approved by the National Council for Statistical Information (CNIS) (visa n°2022AO88ED).

## Conflicts of Interest

The authors declare no conflicts of interest.

## Data Availability

The data that support the findings of this study are not publicly available due to legal and administrative restrictions. However, they can be accessed upon reasonable request and through a formal agreement with the French Ministry of National Education.

## 1

**Table A1:** Descriptive statistics, reliability (Omega ω), and CFA for intrinsic motivation.

Items	Number of items	Omega ω	CFA coef.	*M*	ET
*Factor 1: Intrinsic motivation*	4	0.83			
I do my homework at home, because I like to learn new things.	1		0.70	3.17	1.30
I work in class because I want to learn new things.	2		0.80	3.86	1.15
I work in class because it's pleasant.	3		0.69	3.16	1.24
I try to do well at school, because I am learning things that interest me.	4		0.85	3.67	1.17

*Note:* A least squares estimator was used. Model fit, *χ* ^2^ p < 0.001, RMSEA = 0.096, CFI = 0.94; TLI = 0.90. All coefficients were statistically significant at 99.9%. Omega ω for all items = 0.83. Covariances were added between items 1 and 2, and between items 2 and 4, to account for shared content beyond the latent factor. Items 1 and 2 both reflect intrinsic motivation to learn new things across different contexts (home and school), while items 2 and 4 emphasize learning as a source of personal interest. These conceptual overlaps may lead to residual correlations not captured by the general construct.

Abbreviations: CFA, confirmatory factor analysis; coef., coefficients; RMSEA, root mean square error of approximation; CFI, comparative fi index; TLI, Tucker‐Lewis index.

**Table A2:** Descriptive statistics, reliability (Omega ω), and CFA for extrinsic motivation.

Items	Number of items	Omega ω	CFA coef.	*M*	ET
*Factor 1: Extrinsic motivation*	4	0.68			
I work in class, because I would be ashamed of myself if I did not.	1		0.51	3.06	1.57
I try to do well at school, so that my teachers think I am a good student.	2		0.68	3.64	1.29
I try to do well at school, because I would feel bad about myself if I did not.	3		0.67	3.47	1.38
I try to do well at school, because I think it is important for the job I want to do.	4		0.48	4.43	0.95

*Note:* A least squares estimator was used. Model fit, *χ* ^2^ p < 0.001, RMSEA = 0.096, CFI = 0.94; TLI = 0.90. All coefficients were statistically significant at 99.9%. Omega ω for all items = 0.68.

Abbreviations: CFA, confirmatory factor analysis; coef., coefficients; RMSEA, root mean square error of approximation; CFI, comparative fi index; TLI, Tucker‐Lewis index.

**Table A3:** Descriptive statistics, reliability (Omega ω), and CFA for self‐efficacy.

Items	Number of items	Omega ω	CFA coef.	*M*	*ET*
*Factor 1: Self‐efficacy*	10	0.87			
Are you able to concentrate on your schoolwork?	1		0.75	4.14	0.91
Do you feel able to do your schoolwork when there are more interesting things to do?	2		0.69	3.81	1.10
Are you able to take notes effectively in class?	3		0.62	3.67	1.04
Are you able to organize your schoolwork?	4		0.65	4.09	0.94
Are you able to plan and time your schoolwork?	5		0.67	3.90	1.03
Are you able to memorize what you learn in class and from textbooks?	6		0.60	3.75	0.98
Can you find a place to study without distractions?	7		0.63	3.88	1.13
Are you able to motivate yourself for schoolwork?	8		0.69	3.92	1.01
Can you live up to what your parents expect of you?	9		0.56	4.02	0.99
Can you live up to the expectations of your teachers?	10		0.60	3.88	0.96

*Note:* A least squares estimator was used. Model fit, *χ* ^2^ *p* < 0.001, RMSEA = 0.03, CFI = 0.99, TLI = 0.99. All coefficients were statistically significant at 99.9%. Omega *ω* for all items = 0.85. Covariances were added between items 4 and 5, and between items 9 and 10, based on theoretical and empirical considerations. Items 4 and 5 both refer to students' ability to manage and organize their schoolwork, while items 9 and 10 concern their perceived ability to meet adult expectations. These item pairs are conceptually close, which may account for shared residual variance beyond the latent factor.

Abbreviations: CFA, confirmatory factor analysis; coef., coefficients; RMSEA, root mean square error of approximation; CFI, comparative fi index; TLI, Tucker‐Lewis index.

**Table A4:** Global tests of group differences in terms of sociodemographic characteristics across learner profiles.

Variable	Test	Statistic	*p*	Effect size
Age (years)	ANOVA	*F*(1, 8) = 2006777.55	< 0.001	0.003
Sex	*χ* ^2^	*χ* ^2^(8) = 34.27	< 0.001	0.062
Academic achievement index	ANOVA	*F*(1, 8) = 10631.47	< 0.001	0.803
Numeracy in Grade 7	ANOVA	*F*(1, 8) = 5769.26	< 0.001	0.675
Literacy in Grade 7	ANOVA	*F*(1, 8) = 5093.85	< 0.001	0.675
Family SES index	ANOVA	*F*(1, 8) = 17560.04	< 0.001	0.857
Guardian education level	ANOVA	*F*(1, 8) = 21793.83	< 0.001	0.689
Occupational status	*χ* ^2^	*χ* ^2^(24) = 9512.36	< 0.001	0.596

*Note:* Global differences across the nine learner profiles defined by the cross‐classification of family socioeconomic status (SES: low, mid, high) and academic achievement (ACAD: low, mid, high) were examined using one‐way ANOVA for continuous variables and chi‐square tests of independence for categorical variables. Reported statistics correspond to omnibus tests. Effect sizes are reported as *η* ^2^ for ANOVAs and Cramer's *V* for chi‐square tests. All *p* values are two‐tailed.

**Table A5:** Post hoc Bayesian pairwise comparisons accross learner profiles for intrinsic motivation.

		Prior odds	Posterior odds	BF_10, U_	Error %
SES high × ACAD high	SES high × ACAD low	0.167	47.159	283.189	8.083×10^−5^
	SES high × ACAD mid	0.167	0.553	3.320	0.007
	SES low × ACAD high	0.167	0.021	0.127	0.165
	SES low × ACAD low	0.167	0.126	0.759	0.030
	SES low × ACAD mid	0.167	0.049	0.295	0.075
	SES mid × ACAD high	0.167	0.011	0.065	0.338
	SES mid × ACAD low	0.167	1.054	6.332	0.004
	SES mid × ACAD mid	0.167	0.040	0.241	0.092
SES high × ACAD low	SES high × ACAD mid	0.167	0.057	0.341	0.061
	SES low × ACAD high	0.167	43.407	260.659	9.253×10^−5^
	SES low × ACAD low	0.167	0.126	0.757	0.028
	SES low × ACAD mid	0.167	0.166	0.996	0.021
	SES mid × ACAD high	0.167	1.808	10.855	0.002
	SES mid × ACAD low	0.167	0.037	0.219	0.094
	SES mid × ACAD mid	0.167	0.280	1.682	0.013
SES high × ACAD mid	SES low × ACAD high	0.167	1.227	7.367	0.003
	SES low × ACAD low	0.167	0.010	0.060	0.365
	SES low × ACAD mid	0.167	0.012	0.075	0.288
	SES mid × ACAD high	0.167	0.050	0.297	0.073
	SES mid × ACAD low	0.167	0.009	0.057	0.377
	SES mid × ACAD mid	0.167	0.014	0.087	0.251
SES low × ACAD high	SES low × ACAD low	0.167	0.318	1.912	0.011
	SES low × ACAD mid	0.167	0.170	1.018	0.021
	SES mid × ACAD high	0.167	0.040	0.241	0.087
	SES mid × ACAD low	0.167	1.659	9.961	0.002
	SES mid × ACAD mid	0.167	0.152	0.913	0.023
SES low × ACAD low	SES low × ACAD mid	0.167	0.009	0.053	0.411
	SES mid × ACAD high	0.167	0.020	0.121	0.181
	SES mid × ACAD low	0.167	0.012	0.074	0.296
	SES mid × ACAD mid	0.167	0.009	0.054	0.409
SES low × ACAD mid	SES mid × ACAD high	0.167	0.015	0.091	0.237
	SES mid × ACAD low	0.167	0.016	0.094	0.230
	SES mid × ACAD mid	0.167	0.009	0.054	0.396
SES mid × ACAD high	SES mid × ACAD low	0.167	0.074	0.447	0.049
	SES mid × ACAD mid	0.167	0.013	0.078	0.278
SES mid × ACAD low	SES mid × ACAD mid	0.167	0.019	0.115	0.188

*Note:* Bayesian post hoc comparisons were conducted following the Bayesian ANCOVA on intrinsic motivation. Reported Bayes factors (BF_10_, uncorrected) quantify the evidence in favor of group differences relative to the null hypothesis of no difference. BF_10_ values close to 1 indicate weak or inconclusive evidence, values above 3 indicate moderate evidence, and values above 10 indicate strong to decisive evidence in favor of group differences. Prior odds were set uniformly across comparisons. Posterior odds reflect the updated evidence after observing the data. Error percentages indicate numerical approximation error and are reported for completeness. Age and sex were included as covariates in all models.

**Table A6:** Post hoc Bayesian pairwise comparisons across learner profiles for extrinsic motivation.

		Prior odds	Posterior odds	BF_10, U_	Error %
SES high × ACAD high	SES high × ACAD low	0.167	0.220	1.321	0.016
	SES high × ACAD mid	0.167	0.012	0.072	0.304
	SES low × ACAD high	0.167	0.011	0.067	0.312
	SES low × ACAD low	0.167	1.689	10.142	0.002
	SES low × ACAD mid	0.167	0.021	0.124	0.178
	SES mid × ACAD high	0.167	0.008	0.049	0.447
	SES mid × ACAD low	0.167	0.628	3.772	0.006
	SES mid × ACAD mid	0.167	0.009	0.052	0.425
SES high × ACAD low	SES high × ACAD mid	0.167	0.045	0.269	0.077
	SES low × ACAD high	0.167	0.127	0.760	0.027
	SES low × ACAD low	0.167	0.011	0.069	0.297
	SES low × ACAD mid	0.167	0.026	0.158	0.129
	SES mid × ACAD high	0.167	0.153	0.921	0.023
	SES mid × ACAD low	0.167	0.012	0.073	0.275
	SES mid × ACAD mid	0.167	0.078	0.471	0.045
SES high × ACAD mid	SES low × ACAD high	0.167	0.017	0.104	0.197
	SES low × ACAD low	0.167	0.062	0.370	0.060
	SES low × ACAD mid	0.167	0.010	0.060	0.356
	SES mid × ACAD high	0.167	0.013	0.080	0.270
	SES mid × ACAD low	0.167	0.047	0.285	0.077
	SES mid × ACAD mid	0.167	0.009	0.057	0.379
SES low × ACAD high	SES low × ACAD low	0.167	0.205	1.229	0.018
	SES low × ACAD mid	0.167	0.025	0.147	0.140
	SES mid × ACAD high	0.167	0.012	0.069	0.294
	SES mid × ACAD low	0.167	0.167	1.005	0.021
	SES mid × ACAD mid	0.167	0.013	0.081	0.255
SES low × ACAD low	SES low × ACAD mid	0.167	0.026	0.157	0.140
	SES mid × ACAD high	0.167	0.503	3.021	0.007
	SES mid × ACAD low	0.167	0.009	0.051	0.424
	SES mid × ACAD mid	0.167	0.174	1.047	0.021
SES low × ACAD mid	SES mid × ACAD high	0.167	0.021	0.124	0.174
	SES mid × ACAD low	0.167	0.023	0.138	0.157
	SES mid × ACAD mid	0.167	0.013	0.076	0.286
SES mid × ACAD high	SES mid × ACAD low	0.167	0.290	1.744	0.013
	SES mid × ACAD mid	0.167	0.010	0.060	0.360
SES mid × ACAD low	SES mid × ACAD mid	0.167	0.111	0.668	0.033

*Note:* Bayesian post hoc comparisons were conducted following the Bayesian ANCOVA on extrinsic motivation. Reported Bayes factors (BF_10_, uncorrected) quantify the evidence in favor of group differences relative to the null hypothesis of no difference. BF_10_ values close to 1 indicate weak or inconclusive evidence, values above 3 indicate moderate evidence, and values above 10 indicate strong to decisive evidence in favor of group differences. Prior odds were set uniformly across comparisons. Posterior odds reflect the updated evidence after observing the data. Error percentages indicate numerical approximation error and are reported for completeness. Age and sex were included as covariates in all models.

**Table A7:** Post hoc Bayesian pairwise comparisons across learner profiles for self‐efficacy.

		Prior Odds	Posterior Odds	BF_10, U_	Error %
SES high × ACAD high	SES high × ACAD low	0.167	1.086 × 10^+18^	6.520 × 10^+18^	4.581 × 10^−21^
	SES high × ACAD mid	0.167	4.268 × 10^+10^	2.563 × 10^+11^	1.005 × 10^−13^
	SES low × ACAD high	0.167	0.011	0.069	0.302
	SES low × ACAD low	0.167	7.786 × 10^+38^	4.676 × 10^+39^	6.664 × 10^−42^
	SES low × ACAD mid	0.167	6.617 × 10^+7^	3.973 × 10^+8^	6.303 × 10^−11^
	SES mid × ACAD high	0.167	0.023	0.141	0.156
	SES mid × ACAD low	0.167	1.692 × 10^+31^	1.016 × 10^+32^	3.047 × 10^−34^
	SES mid × ACAD mid	0.167	145,566.933	874,123.416	2.767 × 10^−8^
SES high × ACAD low	SES high × ACAD mid	0.167	7.849	47.134	4.811 × 10^−4^
	SES low × ACAD high	0.167	1.752 × 10^+8^	1.052 × 10^+9^	2.947 × 10^−11^
	SES low × ACAD low	0.167	0.011	0.068	0.302
	SES low × ACAD mid	0.167	24.125	144.872	1.597 × 10^−4^
	SES mid × ACAD high	0.167	2.587 × 10^+11^	1.553 × 10^+12^	1.913 × 10^−14^
	SES mid × ACAD low	0.167	0.012	0.072	0.280
	SES mid × ACAD mid	0.167	8699.103	52,237.752	4.752 × 10^−7^
SES high × ACAD mid	SES low × ACAD high	0.167	1248.180	7495.270	3.241 × 10^−6^
	SES low × ACAD low	0.167	1988.569	11,941.275	1.991 × 10^−6^
	SES low × ACAD mid	0.167	0.011	0.064	0.336
	SES mid × ACAD high	0.167	27,124.810	162,883.362	1.501 × 10^−7^
	SES mid × ACAD low	0.167	175.147	1051.751	2.235 × 10^−5^
	SES mid × ACAD mid	0.167	0.057	0.342	0.064
SES low × ACAD high	SES low × ACAD low	0.167	1.629 × 10^+13^	9.784 × 10^+13^	2.924 × 10^−16^
	SES low × ACAD mid	0.167	88.762	533.012	4.421 × 10^−5^
	SES mid × ACAD high	0.167	0.014	0.082	0.251
	SES mid × ACAD low	0.167	5.797 × 10^+11^	3.481 × 10^+12^	8.495 × 10^−15^
	SES mid × ACAD mid	0.167	5.533	33.224	6.793 × 10^−4^
SES low × ACAD low	SES low × ACAD mid	0.167	14,858.587	89,225.201	2.703 × 10^−7^
	SES mid × ACAD high	0.167	2.291 × 10^+21^	1.376 × 10^+22^	2.131 × 10^−24^
	SES mid × ACAD low	0.167	0.009	0.052	0.417
	SES mid × ACAD mid	0.167	1.137 × 10^+9^	6.830 × 10^+9^	3.736 × 10^−12^
SES low × ACAD mid	SES mid × ACAD high	0.167	634.421	3809.672	6.241 × 10^−6^
	SES mid × ACAD low	0.167	1000.915	6010.453	3.974 × 10^−6^
	SES mid × ACAD mid	0.167	0.020	0.123	0.177
SES mid × ACAD high	SES mid × ACAD low	0.167	1.023 × 10^+18^	6.141 × 10^+18^	4.838 × 10^−21^
	SES mid × ACAD mid	0.167	10.209	61.306	3.741 × 10^−4^

*Note:* Bayesian post hoc comparisons were conducted following the Bayesian ANCOVA on self‐efficacy. Reported Bayes factors (BF_10_, uncorrected) quantify the evidence in favor of group differences relative to the null hypothesis of no difference. BF_10_ values close to 1 indicate weak or inconclusive evidence, values above 3 indicate moderate evidence, and values above 10 indicate strong to decisive evidence in favor of group differences. Prior odds were set uniformly across comparisons. Posterior odds reflect the updated evidence after observing the data. Error percentages indicate numerical approximation error and are reported for completeness. Age and sex were included as covariates in all models.
